# Training effects of affordance judgments in four different settings: towards developing a training battery for affordance judgments

**DOI:** 10.1007/s00221-025-07024-9

**Published:** 2025-03-22

**Authors:** Milena S. Gölz, Isabel Bauer, Lisa Finkel, Cedric Rosati, Andrea Wenzel, Tobias Herrmann, Kenneth F. Valyear, Jennifer Randerath

**Affiliations:** 1 Department of Psychology, University of Konstanz, Constance, Germany; 2 Lurija Institute for Rehabilitation Science and Health Research, Allensbach, Germany; 3 Psychotherapy Training Center, Bodensee (apb), Constance, Germany; 4 School of Psychology and Sport Sciences, Bangor University, Bangor, UK; 5 Clinical Neuropsychology and Neuropsychological Psychotherapy, University of Regensburg, Universitaetsstr. 31, 93053 Regensburg, Germany

**Keywords:** Affordance judgments, Active motor exploration, Outcome feedback, Distractor tasks, Signal detection variables

## Abstract

Training affordance judgments (AJs) across different settings, such as judging whether an object is within reach or an obstacle conquerable, could be meaningful to older adults and neurological patients with diminished judgment behaviors.

The long-term aim is to develop a comprehensive training battery with different types of AJ tasks. The present study used a between-subjects design to evaluate trainability in four different settings. Judgment behavior of 52 healthy young adults was trained (80 trials) in one out of four settings (per trained task N = 13): 1. Reaching horizontally forward for an object, 2. Fitting one hand horizontally into an aperture, 3. Fitting upright under a horizontal barrier, and 4. Stepping over a hurdle. Participants’ judgment performance was assessed pre- versus post-training. Additionally, to assess whether other AJ-based tasks as potential distractors may override training effects, the other three non-trained tasks were presented, and subsequently, judgment performance in the trained task was assessed once more. Accuracy, judgment tendency, and perceptual sensitivity served as dependent variables.

A Friedman Test revealed a main effect of time point in all three variables. Post hoc analyses showed significant improvements in the trained task even after exposure to other AJ tasks.

The results suggest that in young healthy adults, AJs can be trained effectively within different AJ settings, and improvements within one setting can last, even when AJs in other settings are solved in between. Our study provides proof of principle and an important step towards developing a training battery for AJs.

**Supplementary Information:**

The online version contains supplementary material available at 10.1007/s00221-025-07024-9.

## Introduction

Can I cross the street? Can I climb the ladder? Can I reach the highest shelf in the supermarket? These are just three examples of the manifold everyday judgments on action opportunities (affordance judgments, AJs). To avoid injuries, it is important for each individual to assess the fit between their bodily capabilities and the environmental conditions appropriately. Controlled experimental settings investigating performance in such AJs range from reaching (Carello et al. 1989; Gabbard et al. 2005, 2007; Randerath et al. 2021; Randerath and Frey 2016) to overcoming obstacles (Cornus et al. 1999; Daviaux et al. 2014), passing doorways (Hackney and Cinelli 2011) and crossing gaps (Creem-Regehr et al. 2019), to fitting into an aperture (Franchak et al. 2012; Randerath and Frey 2016).

Overall, across different AJ tasks, research has demonstrated that healthy young adults are doing quite well at performing AJs (Cornus et al. 1999; Finkel et al. 2019a; Finkel et al. 2019b; Fitzpatrick et al. 1994; Franchak et al. 2012; Gabbard et al. 2005; Hajnal et al. 2016; Ishak et al. 2008; for an overview see also Finkel et al. 2023). A decline in AJ performance has been reported for advanced age or following brain damage (Finkel et al. 2019a; Randerath et al. 2021). Thus, the development and implementation of a comprehensive training battery to improve performance in AJs across different settings is of great importance. But first controlled studies in healthy, young samples are needed, in order to investigate the general nature and trainability of AJs. To date, some studies have tested training effects in single task-settings and demonstrated improvements after a one-session training (Franchak 2017; Franchak and Somoano 2018; Franchak et al. 2010; Gagnon et al. 2021; Labinger et al. 2018; Randerath and Frey 2016; Wagman 2012; Yasuda et al. 2014; Zhu and Bingham 2010). Training effects have been demonstrated for tasks like doorway passage (Franchak et al. 2010), fitting the hand into apertures (Finkel et al. 2019a; Randerath and Frey 2016) or vertically reaching for objects (Randerath and Frey 2016).

It is important to develop guidelines for designing a training session to elicit desired training effects, as this enhances both the understanding and traceability of the variables involved. Thus far, for AJ trainings, a few supportive factors have been determined: Exploratory movements and feedback format seem to play an important role. For example, Mark et al. (1990) demonstrated that participants wearing 10 cm blocks attached to their feet improved in estimating their own sitting height (altered by the blocks) over time, when they were allowed to locomote between trials (active motor exploration). Stoffregen et al. (2005) replicated these findings using a similar paradigm, additionally pointing out that body sway was associated with learning about maximum sitting height. Beyond the important role that exploratory movements play in providing effective feedback, Franchak and Somoano (2018) as well as Yasuda et al. (2014) stressed another important aspect. They highlighted the pivotal role of the feedback format in enhancing AJs through training. It has been demonstrated that incorporating both, successful and unsuccessful trials, within the training intervention (outcome feedback) is important for its effectiveness (Franchak and Somoano 2018). In particular, in a doorway squeezing task, they found that participants receiving (only) success experience or (only) failure experience did not recalibrate. However, recalibration was possible when both types of feedback experiences were provided.

The nature of locomotion also plays an important role in providing feedback effectively (Franchak 2017; Yasuda et al. 2014). For example, Franchak (2017) showed that participants were unable to recalibrate from mere locomotor experience (active motor exploration). Similar to other studies (Franchak and Adolph 2014; Yasuda et al. 2014), they found that the actual practice of walking through doorways improved AJs. Additionally, the (postural) context was shown to be an important factor for both the perception of affordances (Thomas & Riley 2014) and for improving AJs (Franchak 2017). Thus, for the effectiveness of AJ-training, active motor exploration while experiencing successful versus failed outcomes (outcome feedback) within the tested context of AJs seems to be important.

To date, there have been only a few studies that have investigated feedback training across different affordance tasks. For example, Randerath and Frey (2016) focused on an aperture and a reachability task using a between-subjects design and found an advantageous effect of feedback on judgment performance in both tasks in healthy young adults. For the aperture task, participants were presented with openings that varied in horizontal size, and they had to indicate whether they could fit their hand into the given opening. For the reachability task, participants had to judge whether a presented object would be within their reach. In this study, training was implemented by active motor exploration with outcome feedback. For 80 trials participants were asked to perform the action after indicating their response for a given trial. This allowed exposure to different types of information, including haptic and visual feedback, while experiencing that the hand either does or does not fit into a presented opening or, respectively for the reachability task, whether the object was reachable or not. In addition, participants received acoustic feedback when they managed to perform the action. Moreover, regarding the aperture task, in this sample, it was shown that feedback training within one session for one hand can transfer to the other non-trained hand. Finkel et al. (2019a) unveiled a significant training effect on the accuracy of judgments among both young and elderly adults. This effect was observed both immediately after a training session as well as during a follow-up assessment after one week (5–7 days after training) (Finkel et al. 2019a). Furthermore, recent data on training stroke patients in an aperture task revealed that patients were able to profit from active motor exploration with outcome feedback by attempting to fit their hand into an aperture accompanied by the information about the fit provided by the experimenter in 80 trials (Bauer et al. 2024). At a group level, they performed significantly better following one single training session (Bauer et al. 2024).

It seems important to keep in mind that the described studies involve different types of settings and have different nuances in their type of feedback. There is reason to believe that we should investigate training options for judgment behavior across various types of AJ tasks. The type of task can elicit differential judgment performance. The literature review by Finkel et al. (2023) demonstrated that tasks with proximal boundaries (e.g., judging one’s hand fit into an opening) tend to be judged more conservatively (criterion > 0, participants respond more frequently that the action is not possible, which often is interpreted as a rather cautious behavior) than tasks with distal boundaries (e.g., judging the reachability of a distant object) (Finkel et al. 2023). A very recent study from our lab (Bauer et al. 2025) showed that particularly older participants’ judgment tendencies were significantly more extreme, with stronger under- or overestimations depending on the type of setting. The authors demonstrated significantly more liberal judgments (criterion < 0, participants respond more frequently that the action is possible) in tasks with distal boundaries. Body awareness and attentional alertness correlated with the extent of judgment disparity between setting types.

Our primary long-term objective is to enhance training options for older adults and stroke patients. The goal is to develop a training battery to improve judgment behavior across various types of AJ settings. The current study aims to build the foundation for developing a training battery with which affected persons can be trained in different contexts. We apply active motor exploration with outcome feedback (see ‘Procedure’).

First, we aimed to test whether training effects can be replicated in four different AJ settings:

1. Reachability Task. Previously employed in a recent study by Randerath et al. (2021), this task required the participants to determine whether they could physically reach an object.
2. Aperture Task. This task has also been a subject of investigation in various studies (Gölz et al. 2023; Ishak et al. 2014; Randerath and Frey 2016). Participants were asked to judge whether their hand could fit into an aperture.
3. Fit Under Task. In this task, participants were asked to assess whether they fit under a horizontal barrier (similar to Marcilly and Luyat 2008).
4. Hurdle Task. This task involved participants in evaluating their ability to overcome a hurdle (similar to Petrucci et al. 2016).

These settings include two proximal (Aperture and Fit Under) and two distal tasks (Reachability and Hurdle) according to the definition of Finkel et al. (2023). Task performance was assessed pre- and post-training for three dependent variables: the accuracy of the answers (in %) and two detection theory measures: the sensitivity to discriminate a doable from a non-doable action (measured by the discrimination index d’) and whether the person decides rather liberally or conservatively (judgment tendency measured by criterion c). For the formulas and interpretation of the variables see section ‘Performance variables’. In order to determine the participant’s actual capabilities for each task, initial measurements were conducted before the experiment started (see ‘Tasks’, see for example ‘Reachability Task’).

Second, we aimed to assess whether training effects (active motor exploration with outcome feedback) in one AJ task can last despite being confronted with changing contexts, which would be the case in a training battery. After being assessed and trained in one of the four tasks, the other task settings were implemented as distractor settings. Performance in the trained task was assessed once more thereafter. Another valuable reason for adding distractor settings is presented in everyday life. We are often required to perform various AJ tasks closely together in time. For example, in the supermarket, we must safely navigate our body and a cart through the aisles without bumping into obstacles, and we reach up or bend down to access shopping items in order to put them into the cart. Thus, also to enhance the external validity of the findings, distractor tasks should be integrated into the study design. These distractor tasks served the purpose of examining whether the training-induced effects can persist at a task-specific level over time.

In line with the findings of prior research, which showed significant training effects across multiple AJ tasks (Cole et al. 2013; Finkel et al. 2019a; Randerath and Frey 2016), our hypothesis for the present study postulated that in the four tasks, an improvement takes place after individuals have undergone task-specific training. Furthermore, we assumed that these training-induced enhancements will persist through a follow-up assessment, even when judgments for other AJ settings are solved in between (distractor tasks). Specifically, we anticipated performance enhancements regarding each of the three investigated variables—accuracy (in %), perceptual sensitivity (d’), and judgment tendency (c). An improvement in accuracy would manifest in a higher percentage of correct judgments and an enhancement in perceptual sensitivity towards a higher d’-value. Moreover, an improvement in judgment tendency (c) would be reflected in a value closer to zero, which, in turn, would mean that individuals would have less bias towards liberal or conservative judgments.

## Methods

### Participants

Recruitment of the participants was carried out by posters and flyers distributed in the university building and through the online recruitment software SONA Systems (https://www.sona-systems.com). The study was conducted at the University of Konstanz. Inclusion criteria required right-handedness according to the Edinburgh Handedness Inventory (Salmaso and Longoni 1983), normal or corrected-to-normal vision (assessed through the Snellen chart (Azzam and Ronquillo 2020) and the Lang II Stereo card (Lang 1983; Lang and Lang 1988)), and self-reported absence of psychiatric or neurologic disorders. For the sake of completeness, we also report footedness. 61.5% of the participants were right-footed, while 38.5% of the participants were mixed-footed (analyzed using the Waterloo Footedness Questionnaire—Revised (Elias et al. 1998) based on a lateralization quotient, following the approach of Salmaso and Longoni (1983)).

All participants were naïve to the study’s goals and provided informed written consent. Participation in the study was compensated with study credit (1 per hour) or financially rewarded (10€ per hour).

The project was approved by the ethics committee of the University of Konstanz (#15/2020) and was carried out in accordance with the Declaration of Helsinki. A total of 52 individuals, predominantly students, ranging from 18 to 32 years old (*M*_*age*_ = 23.1 years, *SD*_*age*_ = 3.5 years, 36 female), took part in the four experiments. To avoid sequence effects, greatly expanded length, and related boredom or fatigue effects we decided to use a between-subjects design. Participants were assigned to the different experimental conditions in a quasi-random way (alternation). Demographic variables of the subgroups are described further below as part of the description of the tasks.

### General material

The apparatuses used in the four AJ tasks were custom-built by the scientific workshops at the University of Konstanz. The material and procedure of the Aperture Task and the Reachability Task were adapted from previous studies (Finkel et al. 2019b; Randerath et al. 2021; Randerath and Frey 2016). Across all four tasks, participants responded via button-presses on a response pad (Cedrus, RB540). Participants were asked to press a green button marked “Yes” if they thought they could perform the action and a yellow button marked “No” was pressed for actions estimated as not possible to perform. Judgments were indicated using the right hand.

To control vision during the AJ tasks, throughout the entire experiment, participants wore Plato-goggles (Translucent Technologies Inc.) that could be toggled between opaque and transparent states. They were switched to the opaque state during the initial measurement of participants’ capabilities in the respective tasks (Reachability: maximum arm reachability; Aperture: hand size; Fit Under: body height; Hurdle: maximum stepping-over ability). Also, they were switched to opaque between trials when the adjustments were made to the apparatus, in order to prevent visual feedback. The goggles turned transparent to present a static predefined setting for which participants made their judgment.

SuperLab 5 Software (provided by Cedrus) was used for coding experimental data. For the Aperture Task and the Reachability Task, increments were regulated automatically by a computer-controlled stepper motor. For the Hurdle and the Fit Under Tasks, adjustments were carried out manually by two experimenters. A detailed description of measurements and increments can be found in section ‘Tasks’, ‘Composition of the trials’, and Table 1.

**Table 1:** Composition of the trials [mm]

	„No“-Trials					„Yes“-Trials				
	Filler-trials					Initial value				
Reachability	300	160	80	40	20	0	− 20	− 40	− 80	− 160
Aperture	− 30	− 16	− 8	− 4	− 2	0	2	4	8	16
Fit Under	− 80	− 40	− 20	− 10	− 5	0	5	10	20	40
Hurdle	300	160	80	40	20	0	− 20	− 40	− 80	− 160

*Note*. The initial value 0 reflects the just possible setting (e.g., minimal fit of the hand in the aperture). To prevent an imbalance favoring a correct yes answer more frequently per setting (e.g., for the Aperture Task: 5 yes-trials with 0 mm, +2 mm, +4 mm, +8mm, and +16 mm versus 4 “no”-trials with − 2 mm, − 4 mm, − 8 mm, and − 16 mm), one filler trial per block was added (e.g., for the Aperture Task: − 30 mm). Filler trials were later excluded from the analysis

Participants were instructed to make their judgments as precisely as possible. Participants performed the Fit Under, Hurdle and Reachability Task while standing. For the Aperture Task they sat on a height adjustable chair (see Fig. 2).

### Procedure

**Fig. 1:**
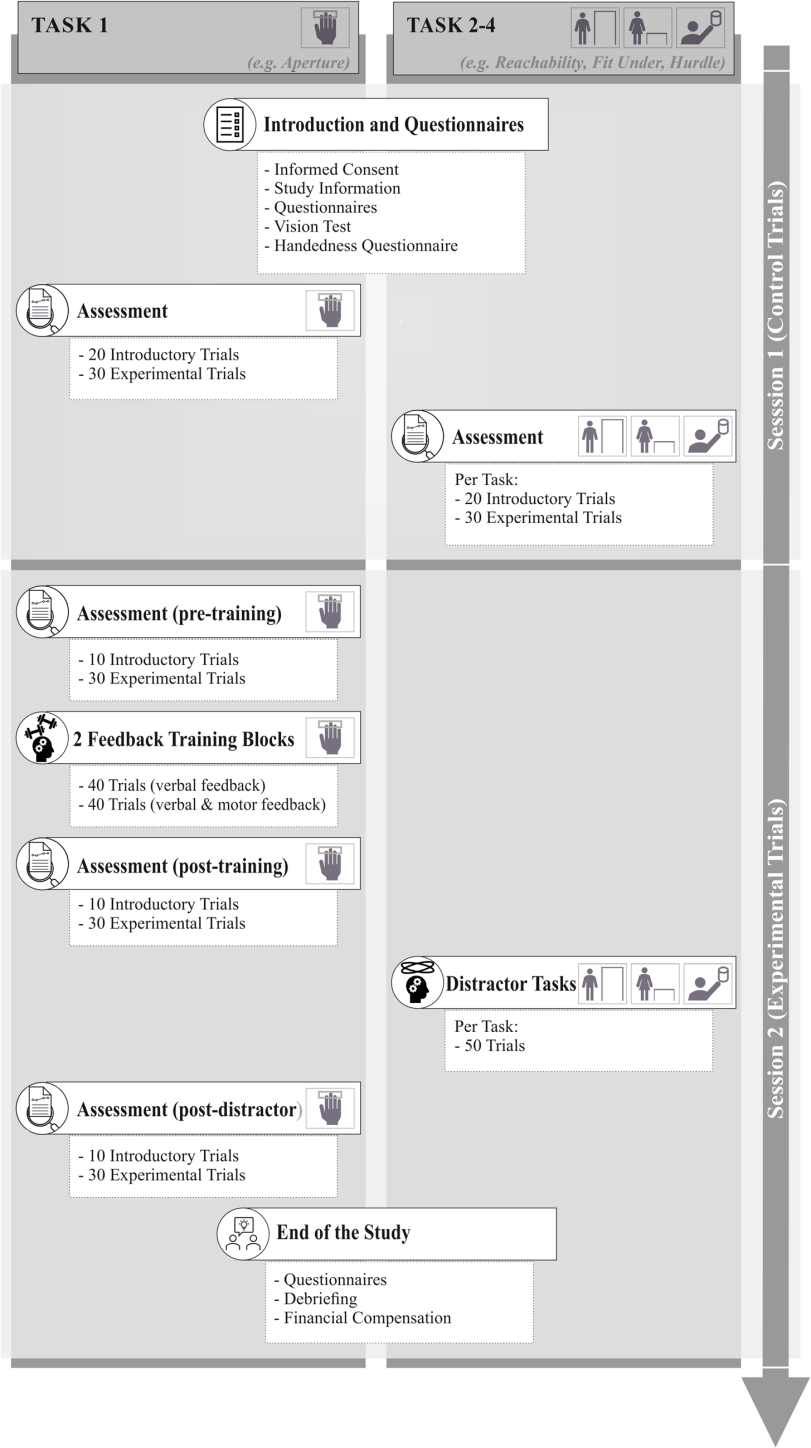
The experimental procedure is exemplarily shown for a person who was trained in the Aperture Task and completed the three other tasks as distractor tasks. *Note.* Participants were assigned to start with one of the four settings which was trained by use of active motor exploration and outcome feedback in the second session

Figure 1 illustrates the study’s procedure. The study was divided into two sessions occurring on two consecutive days. The duration of the first session was approximately 60 min, and the second session (experimental session) took 90 min.

The first session (familiarization and control session, see upper part of Fig. 1) started with participants completing the consent form. This was followed by a vision test and a general questionnaire for demographic information, alongside queries about handed- and footedness. The first session primarily served as a control (mere repetition) session for the AJ tasks. After an introduction to the response pad and Plato-goggles, all participants went through all four AJ tasks. Each of the four AJ tasks consisted of 20 introductory trials (familiarization) and 30 experimental trials (assessment blocks), resulting in a total of 200 trials (4 tasks*(20 introductory trials + 30 experimental trials) = 200 trials) in session 1.

Participants were assigned to one of four conditions, defining which AJ task was trained. For the sake of consistency, the task participants completed first in session 1 was the one they would specifically be tested and trained in on the following day in the experimental session (session 2).

Session 2 (see lower part of Fig. 1) began with a pre-training block comprising 10 introductory trials (first 10 introductory trials of session 1) and 30 experimental trials (assessment block, the same 30 experimental trials as in session 1). This was followed by two blocks of feedback training (2 sets of 40 feedback trials) in the same task. The two training blocks differed in the type of feedback. Across all tasks, in both training blocks, after pressing the button on the response pad, the participants were given verbal feedback, i.e., verbal feedback from the experimenter as to whether the action was actually possible or not. In the second block, in addition to the verbal feedback, the participants were asked to actually try out the action and perform the movement (active motor exploration with outcome feedback). This split of the feedback blocks was done due to time constraints, as especially in the Fit Under and the Hurdle Task the process of walking to the apparatus took up a lot of time. Immediately after the two training blocks, participants engaged in a post-training assessment, encompassing again 10 introductory trials and 30 experimental trials, randomized within blocks. Subsequently, the three remaining AJ tasks were presented as distractor tasks, each involving 50 trials without providing feedback. Participants went through all three tasks, therefore a total of 150 trials. The assignment of the order in which the participants completed the tasks was predefined in the first session.

With the intention of measuring any enduring impact of the feedback, the participants completed a last assessment block with 10 introductory trials and 30 experimental trials of the trained setting. Afterward, participants filled out additional questionnaires, followed by a debriefing.

Please note that session 1 was merely used to familiarize the participants with the experimental setting. Its data was used to check for a potential effect of task exposure or mere repetition on performance. The main analyses testing for training effects only considered data from session 2. The decision to allow for familiarization was made because past studies suggested a need for habituation for unfamiliar conditions (criterion-instability hypothesis (Finkel et al. 2019a)). In this familiarization period, a phase of instability during which a new criterion needs to be built or encoded, is taking place. It is assumed that this process causes variability in the judgment tendency what we wanted to avoid for a clear statement on a potential training effect.

### Tasks

In the following, a description of the four different tasks is provided. In each of them, participants were asked to indicate the feasibility of a specific action. Note that each participant completed all four tasks. However, each participant only received training in one task at a time.

#### Reachability task

**Fig. 2:**
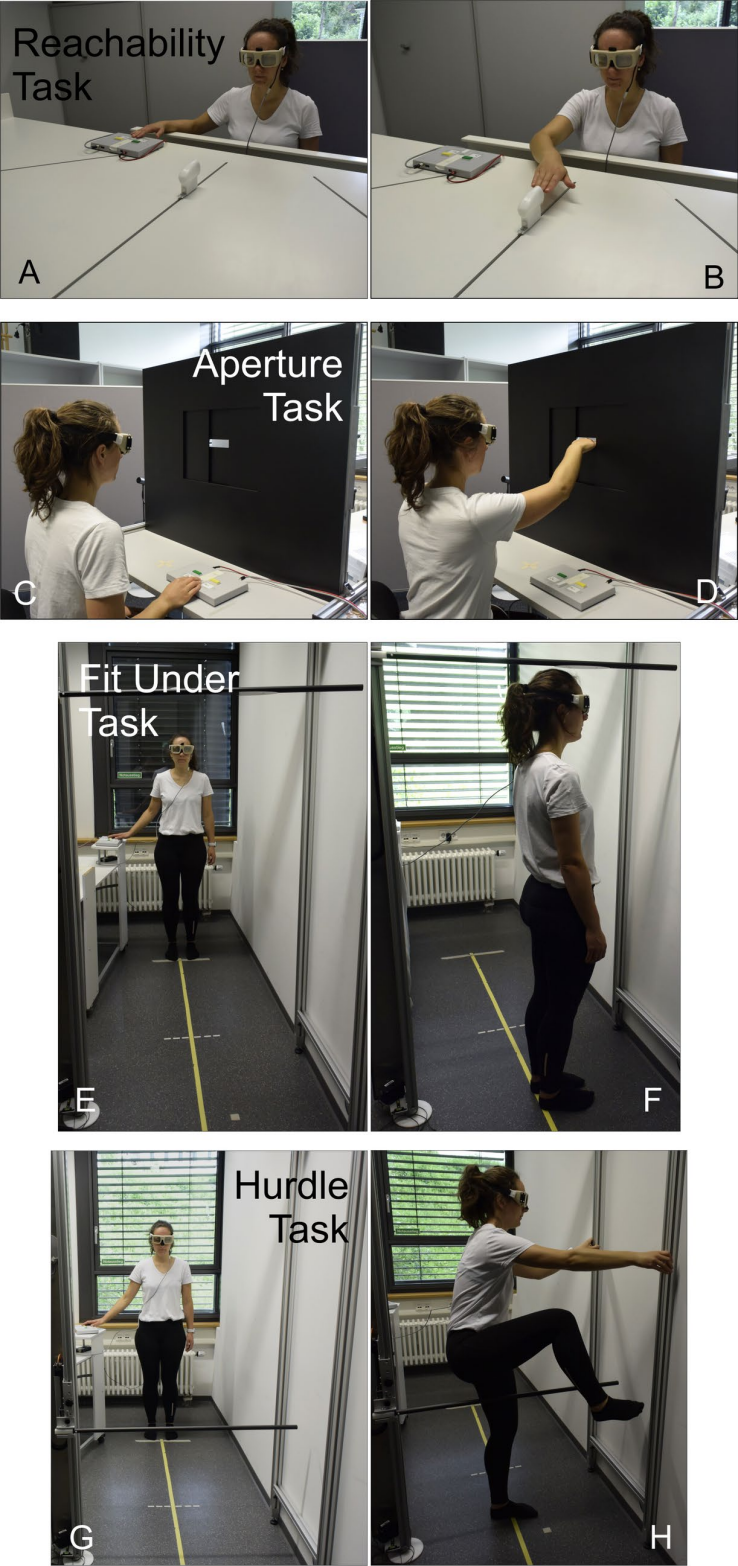
Experimental settings for the four AJ tasks. In the left image, an example of submitting the decision using the response pad is shown. During the assessment only the decision was indicated by the participant. In the right image, an example of a training trial is depicted. During training, first, the decision was indicated, and subsequently, the experimenter verbalized the correct response, either yes or no. In half of the training trials, the participants were also allowed to perform the movement

The setting in the Reachability Task (see Fig. 2 A and B) was initially adapted from Gabbard, Ammar and Lee (Gabbard et al. 2006). It consisted of a height adjustable table with three tracks mounted onto it. On each track, a sled with a small red squared button was presented (see also descriptions in Randerath et al. 2021; Randerath and Frey 2016). In the current setting, these objects could be moved back and forth within the particular track by use of a stepper motor. In the present experiment, only the middle track was utilized. Participants stood, upright in front of the table with their abdomen against the table’s edge to limit forward movement. The table height was adjusted to the participants’ solar plexus. The sled on the middle track was placed centrally in front of the participant.

During the Reachability Task, participants were asked to determine whether a presented object was reachable for their right (dominant) hand. As per task instructions, the object was deemed “within reach” if participants could “successfully touch the red button on the object with the index finger” while standing in front of the apparatus (see Fig. 2A, B). For each trial, the computer-controlled stepper motor moved the sled along the track to distances according to a predefined protocol. Distances were calculated based on the previously measured individual's maximum reachable distance (± 0 trial) with set negative and positive increments (see Table 1). In Fig. 2 A and B, the setup and a training trial of the Reachability Task are depicted. The group of participants who received training in the Reachability Task consisted of 13 individuals (*M*_*age*_ = 21.8 years, *SD*_*age*_ = 2.4 years, 11 female).

Maximum reachability distances were measurable with the help of measurement tapes mounted on the sleds. For the initial measurement, the participants were asked to extend the arm and advance the sled as far as possible while goggles were closed and their view was obstructed. The position of the sled indicated the individual’s maximum reachability distance. This procedure of measuring the critical point from succeeding to failing was carried out three times and on both days in order to take account of day-dependent fluctuations in physical condition (see also Randerath and Frey 2016). The maximum reachability value was used as reference for further settings by the control device which coded trial sequence and controlled the motor for the Reachability Task. For the composition of the trials see ‘Composition of the trials’.

#### Aperture task

For the Aperture Task (see Fig. 2C, D) adapted from Ishak et al. (2008), a custom-built aperture apparatus identical to the one used in previous studies (e.g., Finkel et al. (2019a); Finkel et al. (2019b) or Gölz et al. (2023)) was used. It was made of PVC (black board: 1000 mm length × 850 mm height) and aluminum. Centrally placed, at the participant’s eye level, there was a height and width adjustable rectangular opening.

Participants in the Aperture Task were asked to indicate whether the widest part of their horizontally oriented flat right (dominant) hand could fit through the given horizontally oriented rectangular opening located at eye level (see Fig. 2C, D). During the experiment, following an experimental protocol, two lateral boards on the sides were moved into designated positions by a computer-controlled stepper motor for each trial. The vertical size of the opening was set to the individual’s flat hand’s height, and the horizontal opening sizes varied relative to the participant’s actual hand width (± 0-trial). The experimental setting of the Aperture Task is shown in Fig. 2C, D and the openings covered the increments are described in Table 1. The group of participants who received training in the Aperture Task consisted of 13 individuals (*M*_*age*_ = 23.4 years, *SD*_*age*_ = 3.2 years, 8 female).

Similar to the procedure for the Reachability Task, the actual hand width was measured initially by asking the person, with closed Plato-goggles, to place their flat hand into the opening of the apparatus. The experimenter guided the hand towards the opening. By adjusting the lateral boards, the exact height and width of their hand was determined. This opening represented the maximum hand size (± 0-trial) and was used as reference for further settings that were adjusted in width by the control device. The control device coded trial sequence and controlled the motor for the Aperture Task.

#### Fit under task

The device used in this task (see Fig. 2E, F) included a metal frame that was flush with the wall on one side and had a rod on the other side that could be manually adjusted in height precise to the millimeter. The rod had an oval plate facilitating the size measurement. During the experiment, participants stood at a distance of 150 cm from the device, without wearing shoes (see Fig. 2E, F). In the Fit Under Task, participants were asked to decide whether they could stand upright beneath a presented rod. Following a predefined protocol, the experimenter used an Excel spreadsheet (Microsoft) to calculate the respective height settings which were then manually adjusted using a measuring tape fixed to the apparatus. Due to this manual adjustment, the experiment required the presence of two experimenters throughout. The used increments in the Fit Under Task are described in Table 1. The group of participants who received training in the Fit Under Task consisted of 13 individuals (*M*_*age*_ = 23.3 years, *SD*_*age*_ = 4.3 years, 9 female).

In order to obtain the initial measurement of the actual body height, participants with obstructed view were initially asked to stand under the plate, which was then adjusted. This measurement denoted the individual height (± 0-trial) fitting under the rod.

#### Hurdle task

The apparatus (see Fig. 2 G and H) utilized was the same as the one used in the Fit Under Task—consisting of a metal frame affixed flush with one wall. To the opposite side, a rod measuring 1 m in length could be attached. This rod was manually adjustable in terms of height. The rod was attached in such a way that it fell down when touched. Similar to the Fit Under Task, participants stood at a distance of 150 cm from the device during the experiment, without wearing shoes (see Fig. 2G, H). In the Hurdle Task, participants were asked to make an AJ concerning the capability to surmount the obstacle. Specifically, they were required to assess whether they could climb sideways over the rod and subsequently stand on the floor with both feet without touching the rod. Participants were advised to wear comfortable and well-fitting attire. Similar to the Fit Under Task, the increments for height adjustments were predefined and managed manually using an Excel spreadsheet (Microsoft). The experiment necessitated the involvement of two experimenters. In the context of the Hurdle Task, the ± 0-trials corresponded to the maximum stepping or overcoming height attainable by participants. The maximum stepping-over height is limited by the crotch height. Because all participants (young adults) were able to lift their foot higher than their crotch height, we measured the crotch height as the most appropriate measure for maximal stepping-over height. The used increments can be found in Table 1. The group of participants who received training in the Hurdle Task consisted of 13 individuals (*M*_*age*_ = 23.8 years, *SD*_*age*_ = 3.9 years, 8 female).

The initial measurement was conducted by measuring the crotch height of the individuals standing at the apparatus in an upright position with obstructed view. Experience with this task has shown that it is best suitable that participants adjust the rod to crotch height themselves.

### Composition of the trials

The composition of the trials was based on Randerath and Frey (2016) who carried out two of the four tasks, the Aperture and the Reachability Task, with young, healthy participants in their study. To ensure consistency, both the number of trials and the trial sequence were maintained consistent across the four tasks.

Participants were instructed to assess whether they could still reach an object with their outstretched right arm (Reachability Task), whether they could fit their flat and outstretched right hand through an opening (Aperture Task), whether they could stand upright and straight below the presented height of a rod (Fit Under Task) or whether they can step sideways over the presented height of a rod without dropping it (Hurdle Task). They were asked to provide the answer by pressing a yes or a no button. Participants were instructed to prioritize responding as accurately as possible first, and as quickly as possible second. It was not allowed to actually perform the action.

### Data analysis

Data preparation was performed using Excel (Microsoft). Subsequently, behavioral data underwent analysis through SPSS Statistics 29 (IBM) and JASP (JASP Team, 2024).

#### Performance variables

As dependent variables, different measures were used. First, we examined the accuracy of responses, calculated as the percentage of correct judgments. Additionally, we employed two detection theory variables (see also previous work, e.g., Randerath et al. (2018)), perceptual sensitivity (discriminability index d-prime) and judgment tendency (criterion c) (Green and Swets 1966; Macmillan and Creelman 2004). These variables are derived from Hit and False-Alarm rates. The Hit rate is defined as the ratio of number of hits (person says “yes” if it was a “yes”-trial) to the total number of actual signal occurrences, in our case the number of trials in which “yes” would have been the right answer ($Hit \, Rate= \frac{Number \, of \, Hits}{Number \, of \, Signal \, Occurences}$). In addition, False-Alarm rate means the ratio of the number of False-Alarms (person says “yes” if it was a “no”-trial) to the total number of non-signal occurrences, which are trials in which “no” would have been the correct answer ($False-Alarm \, Rate= \frac{Number \, of \, False-Alarms}{Number \, of \, Non-Signal \, Occurences}$).

Based upon these rates, perceptual sensitivity, representing the capability to perceptually distinguish a fit from a non-fit, was determined using the formula: $d-prime=Z\left(Hit rate\right)-Z(False-Alarm \, rate)$. Higher values indicate a better perceptual discrimination performance.

Furthermore, participants’ judgment tendency was represented by the criterion c which was based on the following formula: $c=-.5*[Z\left(Hit rate\right)+Z\left(False-Alarm \, rate\right)]$. Negative criterion values imply a rather liberal judgment tendency, reflecting a higher frequency of “yes” responses. Conversely, positive criterion values indicate a more conservative judgment tendency, characterized by more “no” responses. For a comprehensive description of the formulas, please see Macmillan and Creelman (2004, pp. 27–31) and for the application of the approach in an earlier study see Randerath and Frey (2016).

In order to analyze the deviation from an ideal criterion (which equals 0), for judgment tendency calculations, we used absolute values of judgment tendency (c). More accurate judgments are represented by higher values for accuracy and perceptual sensitivity, and by values closer to 0 for judgment tendency.

Since the data was not distributed normally in the sample (Shapiro–Wilk Tests revealed significance (p < .05) for accuracy and judgment tendency at the time points post-training and post-distractor), inferential statistics were performed non-parametrically.

#### Effect of mere repetition

We evaluated the potential effect of mere repetition: The data (experimental trials) conducted in Session 1 were compared to the first assessment (pre-training assessment, experimental trials) conducted in Session 2 for each performance variable using Wilcoxon Signed-Rank tests. Results are reported two-tailed (p < .05).

#### Effect of training

Across the four settings, within subjects, we hypothesized a significant improvement following feedback training within the trained tasks for all three measures (accuracy, perceptual sensitivity, and judgment tendency). Additionally, we expected the training effect within the trained task will persist even when the training is followed by distractor tasks requiring AJs. Thus, the main interest of the analyses concentrated on a potential main effect of time point (pre-training/ post-training/ post-distractor).

To test the hypotheses of trainability, three non-parametric analyses of variance (Friedman Tests) were calculated using SPSS Statistics 29 (IBM), one for each of the performance variables accuracy, perceptual sensitivity, and judgment tendency across tasks. Post hoc pairwise comparisons of time points were conducted using Wilcoxon Signed-Rank tests (adjusted *p*-values are provided using the stepwise Holm–Bonferroni procedure (*p*_adj_) to correct for family-wise error rate).

#### Supplemental report of nonparametric results at task level

In order to enable a more nuanced picture, nonparametric Wilcoxon Signed-Rank tests were additionally calculated at task level (see supplementary material S1-S12): pre- versus post-training performance was compared for each task individually. Further, the pre-training performance was compared to the post-distractor performance in order to evaluate if the training effect holds up regardless of the distractor tasks. As the direction of the hypotheses regarding the training effect and the effect of distractor tasks was determined a priori, exact *p*-values were reported one-tailed (*p* < .05). We also provide adjusted *p*-values using the stepwise Holm–Bonferroni procedure (*p*_adj_) to correct for family-wise error rate per task.

## Results

### Effect of mere repetition

Accuracy and the signal detection variables judgment tendency and perceptual sensitivity served as dependent variables representing AJ behavior. Descriptive statistics for all four tasks (Reachability, Aperture, Fit Under, and Hurdle Task) are summarized in Table 3, inferential statistics across tasks for the effect of mere repetition can be found in Table 2. There was no significant change in performance in the variables accuracy, perceptual sensitivity, and judgment tendency from session 1 to session 2.

**Table 2:** Within-subject comparison results across tasks (Wilcoxon Signed-Rank tests) testing for mere repetition effects (experimental trials of session 1 vs. experimental pre-training trials of session 2)

	Z	*p*_*exact*_
Accuracy	− 0.09	.931
Perceptual Sensitivity	− 0.13	.901
Judgment Tendency	− 0.48	.634
Judgment Tendency (absolute value)	− 0.28	.786

### Inferential statistics examining the training effect

**Fig. 3:**
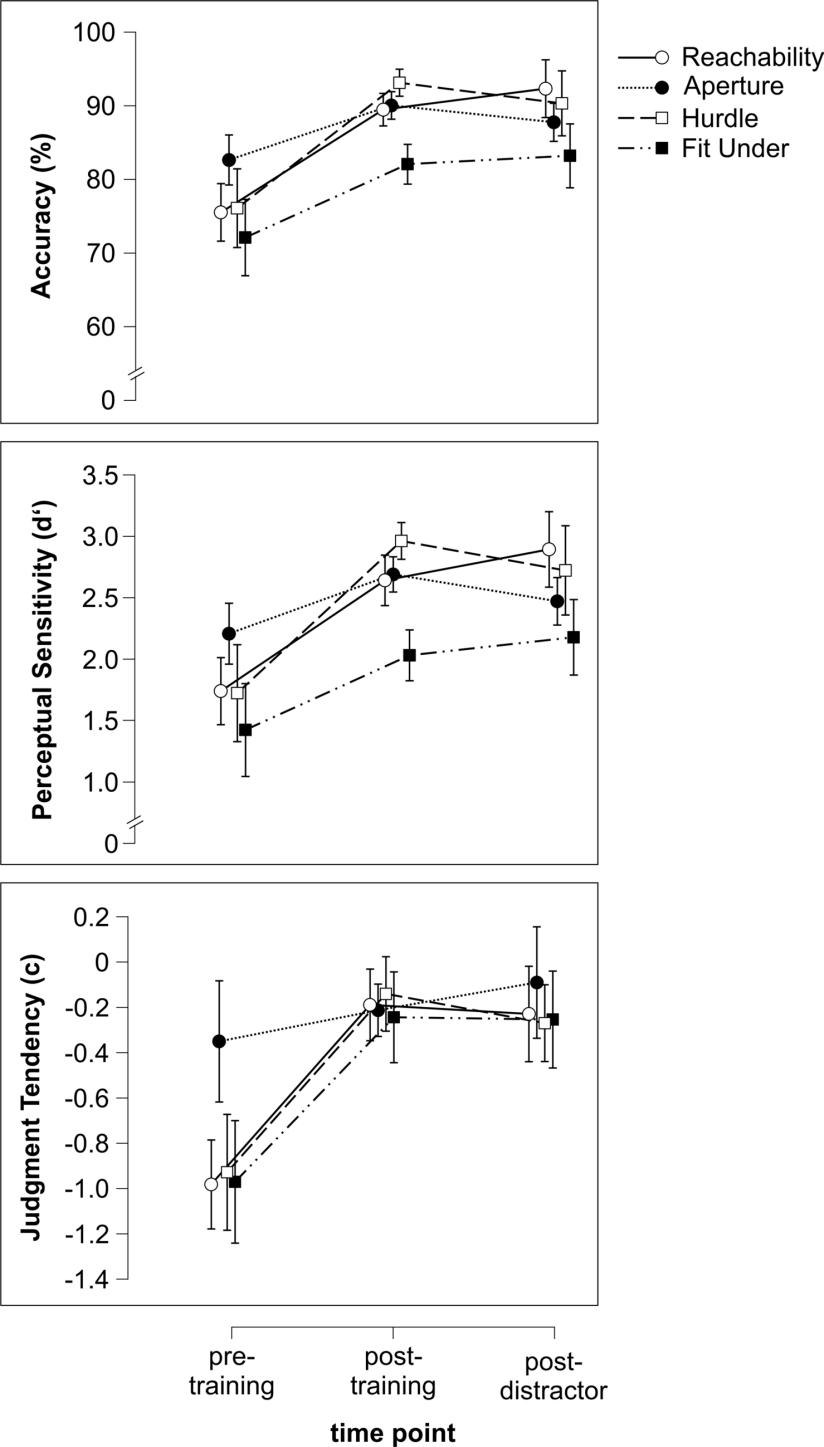
Performance in the four tasks measured across the three time points for accuracy, perceptual sensitivity, and judgment tendency. Error bars represent 95% confidence intervals. Significant improvement from pre-training to post-training and from pre-training to post-distractor assessment was shown for all tasks (see main effect of time point and supplementary material)

To evaluate (the stability of) the training effect for each of the three performance variables, a Friedman Test was conducted with the main aim of examining the effect of time point (pre-training, post-training, and post-distractor). Table 3 summarizes the main effect means and standard deviations. Descriptive plots are depicted in Fig. 3 showing performance in the four tasks across time points for accuracy, perceptual sensitivity, and judgment tendency.

**Table 3:** Main effect means and standard deviations

		Session 1		Pre-training		Post-training		Post-distr	
		*M*	*SD*	*M*	*SD*	*M*	*SD*	*M*	*SD*
Accuracy	Reachability	75.93	11.95	75.50	11.57	89.46	6.21	92.31	5.75
	Aperture	81.77	9.50	82.62	7.91	90.03	5.93	87.75	6.12
	Fit Under	73.50	5.83	72.08	7.50	82.05	7.54	83.19	9.64
	Hurdle	74.36	11.56	76.07	13.74	93.12	6.08	90.31	5.56
	across all tasks	76.39	10.24	76.57	10.90	88.66	7.50	88.39	7.59
Perceptual Sensitivity	Reachability	1.76	0.89	1.74	0.83	2.64	0.51	2.89	0.53
	Aperture	2.11	0.58	2.21	0.54	2.69	0.49	2.47	0.52
	Fit Under	1.57	0.41	1.42	0.49	2.03	0.64	2.18	0.71
	Hurdle	1.59	0.82	1.72	1.01	2.96	0.58	2.72	0.45
	across all tasks	1.75	0.72	1.77	0.78	2.58	0.64	2.57	0.61
Judgment Tendency	Reachability	− 0.92	0.59	− 0.98	0.45	− 0.19	0.45	− 0.23	0.33
	Aperture	− 0.38	0.66	− 0.35	0.69	− 0.21	0.44	− 0.09	0.44
	Fit Under	− 0.54	0.72	− 0.97	0.42	− 0.24	0.49	− 0.26	0.52
	Hurdle	− 1.09	0.41	− 0.93	0.56	− 0.14	0.29	− 0.27	0.46
	across all tasks	− 0.73	0.66	− 0.81	0.59	− 0.20	0.41	− 0.21	0.43

The results for the three performance variables looked similar: there was a significant main effect of time point, indicating that test scores changed significantly (see Table 4). Pairwise comparisons of time points (see Table 4) showed significantly improved scores: higher accuracy and perceptual sensitivity values, and a judgment tendency (absolute values) closer to zero for post- compared to pre-training. The improvement was also observed in post-distractor tasks compared to pre-training, and no significant difference was found when comparing the performance post-training to post-distractor tasks.

**Table 4:** Results of Friedman Tests and post hoc comparisons for performance across tasks for the three time points

**Friedman Tests**			χ²			df				*p*		
Accuracy			38.18			2				<.001		
Perceptual Sensitivity			29.89			2				<.001		
Judgment Tendency (absolute values)			38.56			2				<.001		
**Post hoc pairwise comparisons**		*M*_dn Variable 1_	SD_Variable1_	*M*_dn Variable 2_		SD _Variable2_	Z	p_exact_		p_adj_	*r*	
**Accuracy**												
Pre-training	Post-training	77.78	10.90	88.89		7.50	-5.64	<.001		<.001	-0.55	
Pre-training	Post-distractor	77.78	10.90	88.89		7.59	-4.92	<.001		<.001	-0.48	
Post-training	Post-distractor	88.89	7.50	88.89		7.59	-0.16	.875		.875	-0.02	
**Perceptual Sensitivity**												
Pre-training	Post-training	1.74	0.78	2.55		0.64	-5.48	<.001		<.001	-0.54	
Pre-training	Post-distractor	1.74	0.78	2.55		0.61	-4.56	<.001		<.001	-0.45	
Post-training	Post-distractor	2.55	0.64	2.55		0.61	-0.27	.793		>.999	-0.03	
**Judgment Tendency (absolute values)**												
Pre-training	Post-training	0.94	0.45	0.35		0.26	-5.43	<.001		<.001	-0.53	
Pre-training	Post-distractor	0.94	0.45	0.43		0.24	-4.89	<.001		<.001	-0.48	
Post-training	Post-distractor	0.35	0.26	0.43		0.24	-0.91	.370		>.999	-0.09	

## Discussion

Our long-term objective is to expand training options for older adults and stroke patients by creating a comprehensive training program aimed at improving affordance judgment (AJ) behavior across different settings. This study sought to lay the groundwork for developing a versatile AJ training battery that enables individuals to be trained effectively in multiple contexts. In the current study, we evaluated whether training effects based on active motor exploration with outcome feedback can be consistently replicated across four distinct settings. Additionally, we investigated whether the training effects achieved in one AJ task can persist despite exposure to varying contexts, as expected in a comprehensive training program. Our results confirmed that training can be effective, and improvements can persist even when participants engaged in distinct AJ-based distractor tasks. The outcome that different types of AJs can be trained effectively holds promise for individuals whose AJ performance is altered or impaired, such as the elderly or stroke patients (Finkel et al. 2019a, 2019b; Randerath et al. 2021). In the following, we will discuss implications and identify potential future steps that could facilitate the implementation of AJ training to benefit target groups.

Previous studies demonstrated advantageous training effects of active motor exploration and outcome feedback in various AJ tasks, including reaching (Randerath and Frey 2016), doorway passage (Franchak et al. 2010), and aperture tasks (Finkel et al. 2019a; Gölz et al. 2023). The present results add to this research by showing that different tasks can be effectively trained. Notably, we assessed the effects of mere repetition and did not find any significant changes in participants’ performance across tasks when they only were allowed to judge but did not receive any feedback. This suggests that repetition of judgments alone does not improve performance. Across tasks, training effects obtained within one session, including feedback, were demonstrated for each of the three performance variables. Participants showed substantial improvements in accuracy, perceptual sensitivity, and judgment tendency from pre-training to post-training in the trained task. These improvements lasted even after introducing other AJ tasks.

Our post hoc analysis elucidating training effects for each individual task demonstrated one exception: the Aperture Task (see supplementary material). The group of participants who received training in the Aperture Task experienced significant improvements in accuracy, perceptual sensitivity, and judgment tendency from pre-training to post-training. However, after introducing distractor tasks, only judgment tendency showed stable improvement, while accuracy and perceptual sensitivity did not. The partial lack of maintenance of the training effect in the Aperture Task over distractor tasks could potentially be attributed to ceiling effects with high-performance baseline levels and limited potential for strong training effects. Previous studies using the Aperture Task (Gölz et al. 2023; Randerath and Frey 2016) have already shown that young and healthy participants frequently make highly accurate judgments right from the beginning, even without training. In our current study, across tasks, the sample of young healthy adults showed the highest performance levels in the Aperture Task before training, with accuracy at approximately 80%, perceptual sensitivity at 2.08, and judgment tendency at -0.60. This contrasts with older adults or stroke patients, who generally have lower baseline performance in this task (Bauer et al. 2024; Finkel et al. 2019a; Randerath et al. 2018). Thus, while the high baseline in our sample may have limited our ability to detect lasting training gains for accuracy and perceptual sensitivity after the distractor tasks in the Aperture Task, other samples with lower baselines may demonstrate more training gains (Bauer et al. 2024).

Our results demonstrate that brief feedback-driven learning can improve decisions about action opportunities in different AJ settings. These improvements persist even though participants encountered other, non-trained AJ settings. Our results line up with a range of studies successfully demonstrating that goal-directed action planning can be learned and even locked to very specific settings (Cole 2008; Cothros et al. 2009; Li et al. 2009; Wolpert et al. 2011). For example, Li et al. (2009) demonstrate that participants lifting objects of similar size successfully form an association between an individual object’s color and its manipulated weight. Participants incorporate this association into grip force programming and retrieve object-specific grip force scaling according to its associated weight. Many different types of studies support that young, healthy adults’ perceptual-motor system is specifically trainable despite changing sensorimotor contexts. Healthy young adults quickly adapt their judgments to changed body properties, for example, overestimation of reachability is enhanced after using a tool (Bourgeois et al. 2014; Luyat et al. 2024), or when being equipped with a hand splint judgments are adjusted to its new boundaries (Finkel et al. 2019b). Such findings support a flexible and dynamic perception–action system highly adaptive to its environment claimed by several influential theories [e.g., Theory of Event Coding: Hommel (2015); Hommel et al. (2001); Hommel and Wiers (2017) or affordance competition hypothesis: Cisek (2007); Cisek and Kalaska (2010)]. Our environment often presents a vast array of stimuli and action opportunities, while demands for quick, adaptive, and precise decisions vary between settings. From an evolutionary perspective, it may, for example, make sense to mistake a stick for a snake but not a snake for a stick when running through environments where snakes may be present (Johnson et al. 2013).

Our study serves as a proof of concept for further research into the trainability of the presented AJs within task batteries. The long-term goal is to develop targeted training programs that support affected groups, such as stroke patients and older adults, in improving their ability to navigate everyday challenges more effectively.

### Limitations and outlook

Some limitations of the current study need to be mentioned. First, it is important to keep in mind that the tested sample only included right-handed subjects. Follow-up studies may systematically consider handedness as well as footedness as variables of interest in order to explore their potential influence on AJs. Second, our participants received mainly training based on active motor exploration with outcome feedback specific to the respective setting. At this point, we cannot disentangle the contribution of the different types of information participants received by this approach. Third, while we assigned the participants to the respective group in a quasi-random way, we cannot fully exclude that the chosen between-subjects design may have added inadvertent variability due to individual differences per group. Still, at this stage, points in favor of a within-subjects design were outweighed by the disadvantages (e.g., sequence effects, greatly expanded length, and related boredom or fatigue effects). Also, differences between groups were not of major interest for our analyses. Fourth, one notable practical issue arose during task implementation concerning the measurement of actual abilities in tasks with more degrees of freedom, such as stepping-over height in the Hurdle Task. Alternatives such as automated measurements could be considered, for example, using laser measuring instruments could improve and simplify procedures. Fifth, this study includes a homogeneous sample of primarily young, healthy, predominantly female, and highly educated participants. Consequently, interpreting our results should be mindful of this limitation (Henrich et al. 2010).

Future research should also consider studying other groups, and in particular those with lower judgment performance, to increase knowledge about the applicability across diverse samples. For further development and evaluation of an AJ training battery, it would be desirable to apply, for example, the current set to affected samples such as the elderly or stroke patients in order to test whether they also benefit from this task-specific training. Promising results were already observed in a group of stroke patients who showed significant performance increases during training as well as post-training in one of the tasks, the Aperture Task (Bauer et al. 2024). In addition, it could be beneficial to save effort, space (no more large apparatuses) and costs to implement the tasks in a virtual environment. It would then be possible for affected persons to train independently and in a safe environment (gamification approaches would also be conceivable). One of our recent studies applying the Aperture Task virtually has demonstrated that performance variables became increasingly more similar between physical and virtual environments after VR training (Gölz et al. 2023).

## Conclusion

We studied the training potential of affordance judgments across four distinct settings and assessed the persistence of training effects when participants encountered additional AJ tasks. The findings reveal significant improvements in trained AJs, with persisting training gain even after participants engaged in other AJ tasks. These results extend existing research on AJ performance, highlighting the efficacy of AJ training and offering a platform for further refinement of future training protocols.

## Supplementary Information

Below is the link to the electronic supplementary material.

Supplementary file1 (XLSX 16 KB)

Supplementary file1 (DOCX 2369 KB)

## Data Availability

The data for all experiments will be made available in the supplementary upon acceptance.
