# Supplementary material for: Training effects of affordance judgments in four different settings: towards developing a training battery for affordance judgments
Source: Exp Brain Res. 2025 Mar 22;243(4):98. doi: 10.1007/s00221-025-07024-9 (PMC11929631; doi:10.1007/s00221-025-07024-9)
Supplement: Supplementary file 2 — Supplementary file1 (DOCX 2369 KB) [file 221_2025_7024_MOESM2_ESM.docx]

S1 Text. Inferential statistics examining the training effect (Wilcoxon Signed-Rank tests): Reachability Task

Regarding the Reachability Task, performance improved significantly from pre- to post-training regarding all three variables, accuracy, perceptual sensitivity, and judgment tendency. Furthermore, the comparison of pre-training performance to the performance post-distractor exposition still revealed a significant improvement in all three variables (accuracy, perceptual sensitivity, and judgment tendency). S3 Figure displays accuracy, perceptual sensitivity, and judgment tendency performance in the Reachability Task across different time points: pre-training, the two training blocks, post-training, and post-exposition to the distractor tasks. Descriptive and inferential statistics are listed in S2 Table.

S2 Table. Descriptive and inferential statistics of accuracy, perceptual sensitivity, and judgment tendency in the Reachability Task for the three time points.

|  | **pre-training** | | **post-training** | | **effect of training (pre- vs. post-training)** | | | | **post-distractor** | | **effect of distractor tasks (pre-training vs. post-distractor)** | | | |
| --- | --- | --- | --- | --- | --- | --- | --- | --- | --- | --- | --- | --- | --- | --- |
| **Variable** | ***M_dn_*** | ***SD*** | ***M_dn_*** | ***SD*** | ***Z*** | ***p_exact_*** | ***p_adj_*** | ***r*** | ***M_dn_*** | ***SD*** | ***Z*** | ***p_exact_*** | ***p_adj_*** | ***r*** |
| accuracy (%) | 74.07 | 11.57 | 88.89 | 6.21 | -3.10 | <.001 | .002 | .607 | 92.59 | 5.75 | -2.99 | <.001 | .001 | .586 |
| perceptual sensitivity (d-prime) | 1.68 | 0.83 | 2.55 | 0.51 | -3.04 | <.001 | .002 | .596 | 2.89 | 0.53 | -2.98 | <.001 | .001 | .585 |
| judgment tendency (criterion c) | -1.04 1.04 | 0.45 0.45 | -0.07 0.41 | 0.45 0.28 | -3.11 | <.001 | .001 | .610 | -0.24 0.24 | 0.33 0.25 | -2.83 | .001 | .001 | .554 |

*Note.* *p_adj_* = Holm-Bonferroni adjusted p-values. *Please note.* The second line for each session of the variable judgment tendency indicates the *M_dn_* and *SD* for the absolute judgment tendency values (absolute difference to an ideal criterion of 0). For inference statistics regarding judgment tendency only absolute values were used.


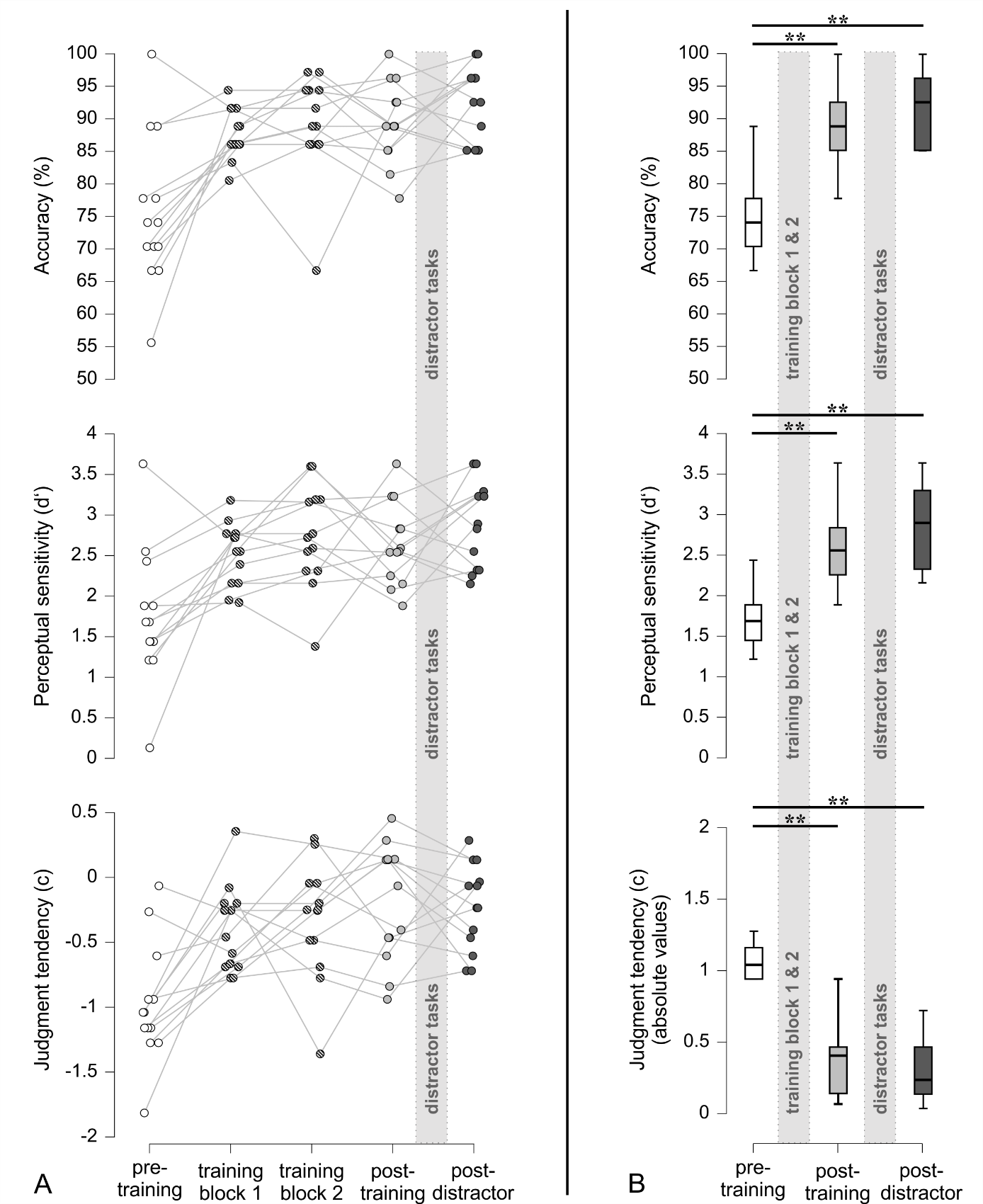


**S3 Figure.** Distribution of individual data and boxplots for accuracy [%], perceptual sensitivity (d-prime), and judgment tendency (criterion) performance in the Reachability Task (N=13).

(A) Distribution of the individual data per person across time points for accuracy, perceptual sensitivity, and judgment tendency. (B) Boxplots representing performance at a certain time point (pre-training, post-training, post-distractor). The significance of time point comparisons is indicated. Please note. For judgment tendency, we used absolute values to analyze the deviation from an ideal criterion (which equals 0): The closer to zero the better the performance. Note. * p < .05, ** p < .01 (after Holm–Bonferroni adjustment).

S4 Text. Inferential statistics examining the training effect (Wilcoxon Signed-Rank tests): Aperture Task

Results revealed a significant improvement from pre- to post-training in regard to each of the three variables (accuracy, perceptual sensitivity, and judgment tendency). When comparing pre-training to the post-distractor assessment, the training effect yield no significance in accuracy and perceptual sensitivity, but judgment tendency. S6 Figure displays accuracy, perceptual sensitivity and judgment tendency performance in the Aperture Task across the three time points in boxplots, parallel to S3 Figure for the Reachability Task. Descriptive and inferential statistics are listed in S5 Table.

S5 Table. Descriptive and inferential statistics of accuracy, perceptual sensitivity, and judgment tendency in the Aperture Task for the three time points.

|  | **pre-training** | | **post-training** | | **effect of training (pre- vs. post-training)** | | | | **post-distractor** | | **effect of distractor tasks (pre-training vs. post-distractor)** | | | |
| --- | --- | --- | --- | --- | --- | --- | --- | --- | --- | --- | --- | --- | --- | --- |
| **Variable** | ***Mdn*** | ***SD*** | ***Mdn*** | ***SD*** | ***Z*** | ***P_exact_*** | ***p_adj_*** | ***r*** | ***Mdn*** | ***SD*** | ***Z*** | ***P_exact_*** | ***p_adj_*** | ***r*** |
| accuracy (%) | 81.48 | 7.91 | 92.59 | 5.93 | -2.70 | .003 | .018 | .529 | 88.89 | 6.12 | -1.80 | .041 | .082 | .353 |
| perceptual sensitivity (d-prime) | 2.08 | 0.54 | 2.83 | 0.49 | -2.55 | .004 | .020 | .501 | 2.55 | 0.52 | -1.10 | .151 | .151 | .215 |
| judgment tendency (criterion c) | -0.61 0.66 | 0.69 0.33 | -0.41 0.47 | 0.44 0.22 | -2.38 | .007 | .028 | .466 | -0.20 0.31 | 0.44 0.18 | -2.34 | .008 | .024 | .459 |

*Note.* *p_adj_* = Holm-Bonferroni adjusted p-values. *Please note.* The second line for each session of the variable judgment tendency indicates the *M_dn_* and *SD* for the absolute judgment tendency values (absolute difference to an ideal criterion of 0). For inference statistics regarding judgment tendency only absolute values were used.


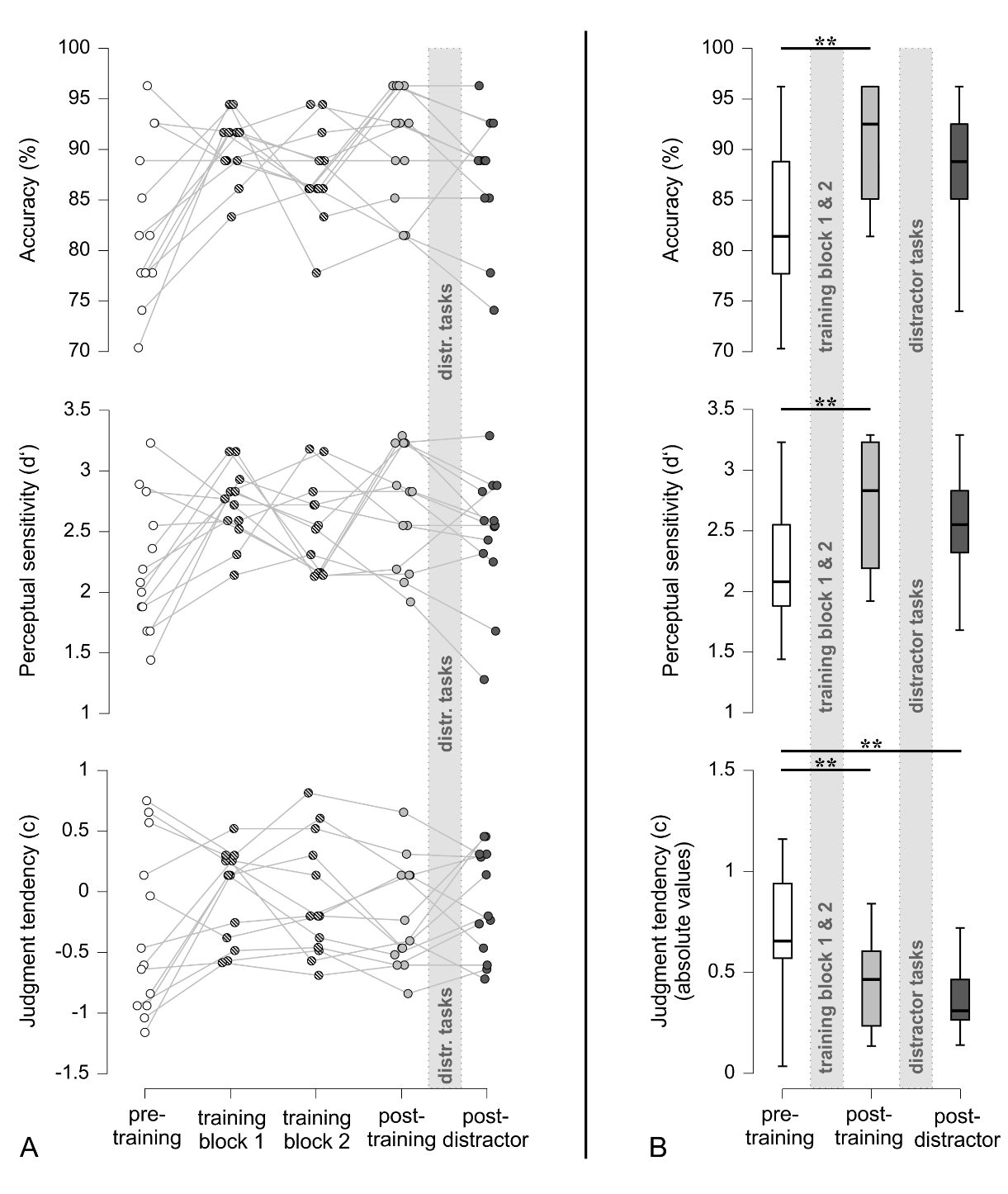


S6 Figure. Distribution of individual data and boxplots for accuracy [%], perceptual sensitivity (d-prime), and judgment tendency (criterion) performance in the Aperture Task (N=13).

(A) Distribution of the individual data per person across time points for accuracy, perceptual sensitivity, and judgment tendency. (B) Boxplots representing performance at a certain time point (pre-training, post-training, post-distractor). The significance of time point comparisons is indicated. Please note. For judgment tendency, we used absolute values to analyze the deviation from an ideal criterion (which equals 0): The closer to zero the better the performance. Note. * p < .05, ** p < .01 (after Holm–Bonferroni adjustment).

S7 Text. Inferential statistics examining the training effect (Wilcoxon Signed-Rank tests): Fit Under Task

Results showed a significant improvement from pre- to post-training in regard to all three performance variables (accuracy, perceptual sensitivity, and judgment tendency). A similar picture emerged when comparing pre-training to post-distractor assessment. Participants improved significantly in accuracy, perceptual sensitivity, as well as judgment tendency. In the same way as S3 Figure and S6 Figure for the Reachability and the Aperture Task, S9 Figure displays the performance variables in the Fit Under Task across the three time points represented in boxplots. Descriptive and inferential statistics are listed in S8 Table.

S8 Table. Descriptive and inferential statistics of accuracy, perceptual sensitivity, and judgment tendency in the Fit Under Task for the three time points.

|  | **pre-training** | | **post-training** | | **effect of training (pre- vs. post-training)** | | | | **post-distractor** | | **effect of distractor tasks (pre-training vs. post-distractor)** | | | |
| --- | --- | --- | --- | --- | --- | --- | --- | --- | --- | --- | --- | --- | --- | --- |
| **Variable** | ***Mdn*** | ***SD*** | ***Mdn*** | ***SD*** | ***Z*** | ***P_exact_*** | ***p_adj_*** | ***r*** | ***Mdn*** | ***SD*** | ***Z*** | ***P_exact_*** | ***p_adj_*** | ***r*** |
| accuracy (%) | 70.37 | 7.50 | 81.48 | 7.54 | -2.36 | .008 | .031 | .462 | 85.19 | 9.64 | -2.28 | .010 | .021 | .447 |
| perceptual sensitivity (d-prime) | 1.44 | 0.49 | 1.92 | 0.64 | -2.06 | .019 | .019 | .404 | 2.15 | 0.71 | -2.34 | .009 | .026 | .459 |
| judgment tendency (criterion c) | -0.94 0.94 | 0.42 0.42 | -0.35 0.46 | 0.49 0.30 | -2.41 | .007 | .034 | .473 | -0.41 0.47 | 0.52 0.27 | -2.48 | .005 | .031 | .487 |

*Note.* *p_adj_* = Holm-Bonferroni adjusted p-values. *Please note.* The second line for each session of the variable judgment tendency indicates the *M_dn_* and *SD* for the absolute judgment tendency values (absolute difference to an ideal criterion of 0). For inference statistics regarding judgment tendency only absolute values were used.


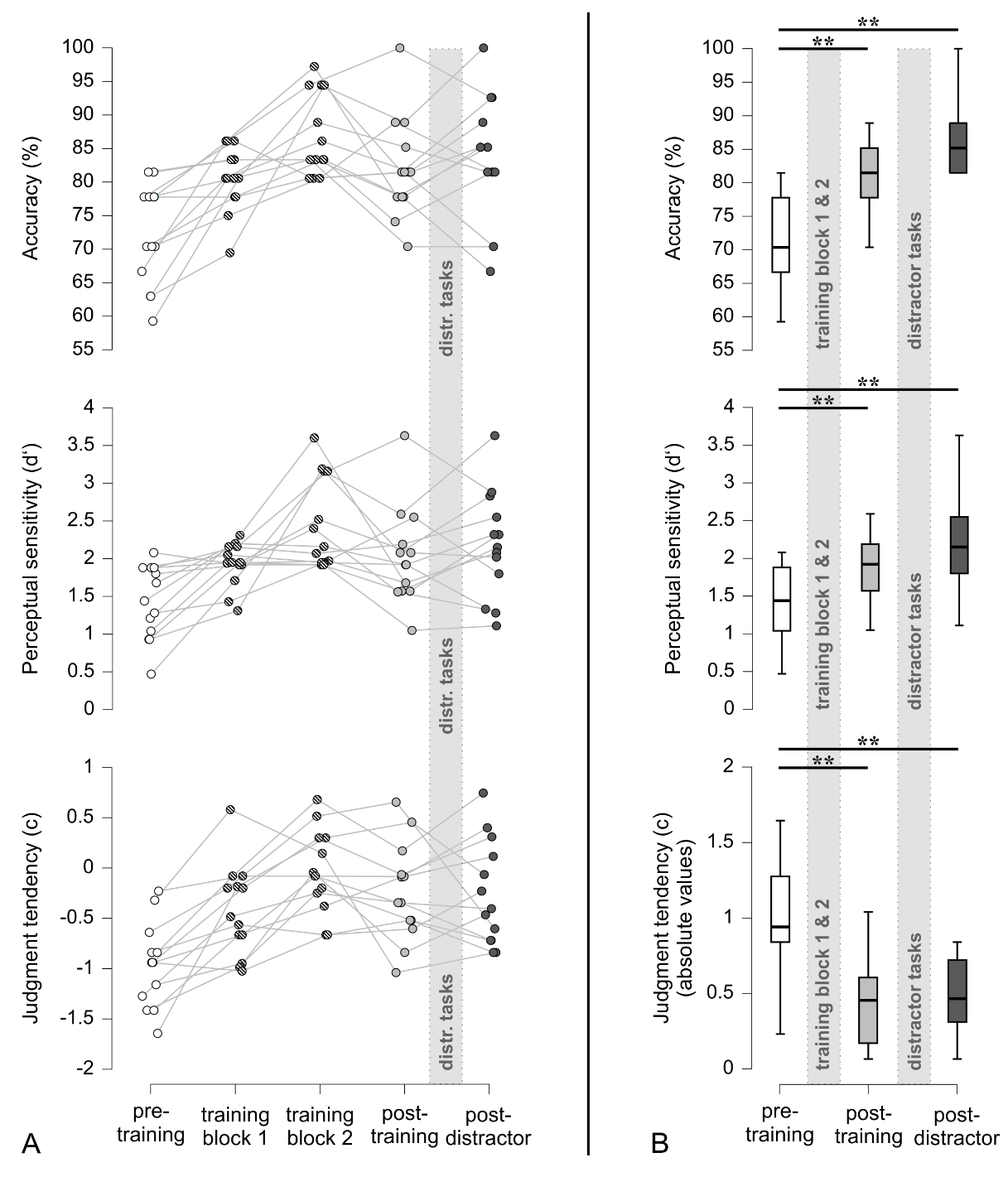


S9 Figure. Distribution of individual data and boxplots for accuracy [%], perceptual sensitivity (d-prime), and judgment tendency (criterion) performance in the Fit Under Task (N=13).

(A) Distribution of the individual data per person across time points for accuracy, perceptual sensitivity, and judgment tendency. (B) Boxplots representing performance at a certain time point (pre-training, post-training, post-distractor). The significance of time point comparisons is indicated. Please note. For judgment tendency, we used absolute values to analyze the deviation from an ideal criterion (which equals 0): The closer to zero the better the performance. Note. * p < .05, ** p < .01 (after Holm–Bonferroni adjustment).

S10 Text. Inferential statistics examining the training effect (Wilcoxon Signed-Rank tests): Hurdle Task

Results revealed a significant improvement in performance from pre- to post-training within all three variables (accuracy, perceptual sensitivity, and judgment tendency). Moreover, when comparing pre-training to post-distractor assessment, the improvement was preserved significantly regarding each of the variables: accuracy, judgment tendency, and perceptual sensitivity. S12 Figure displays performance variables for each time point in boxplots, similar to the other experiments (S3 Figure, S6 Figure, and S9 Figure). Descriptive and inferential statistics are listed in S11 Table.

S11 Table. Descriptive and inferential statistics of accuracy, perceptual sensitivity, and judgment tendency in the Hurdle Task for the three time points.

|  | **pre-training** | | **post-training** | | **effect of training (pre- vs. post-training)** | | | | **post-distractor** | | **effect of distractor tasks (pre-training vs. post-distractor)** | | | |
| --- | --- | --- | --- | --- | --- | --- | --- | --- | --- | --- | --- | --- | --- | --- |
| **Variable** | ***Mdn*** | ***SD*** | ***Mdn*** | ***SD*** | ***Z*** | ***P_exact_*** | ***p_adj_*** | ***r*** | ***Mdn*** | ***SD*** | ***Z*** | ***P_exact_*** | ***p_adj_*** | ***r*** |
| accuracy (%) | 77.78 | 13.74 | 92.59 | 6.08 | -2.94 | <.001 | .003 | .577 | 88.89 | 5.56 | -2.63 | .003 | .010 | .515 |
| perceptual sensitivity (d-prime) | 1.68 | 1.01 | 2.88 | 0.58 | -2.98 | <.001 | .003 | .585 | 2.59 | 0.45 | -2.41 | .007 | .013 | .473 |
| judgment tendency (criterion c) | -0.84 0.84 | 0.56 0.56 | -0.07 0.24 | 0.29 0.19 | -2.90 | <.001 | .003 | .569 | -0.47 0.47 | 0.46 0.25 | -2.20 | .013 | .013 | .432 |

*Note.* *p_adj_* = Holm-Bonferroni adjusted p-values. *Please note.* The second line for each session of the variable judgment tendency indicates the *M_dn_* and *SD* for the absolute judgment tendency values (absolute difference to an ideal criterion of 0). For inference statistics regarding judgment tendency only absolute values were used.


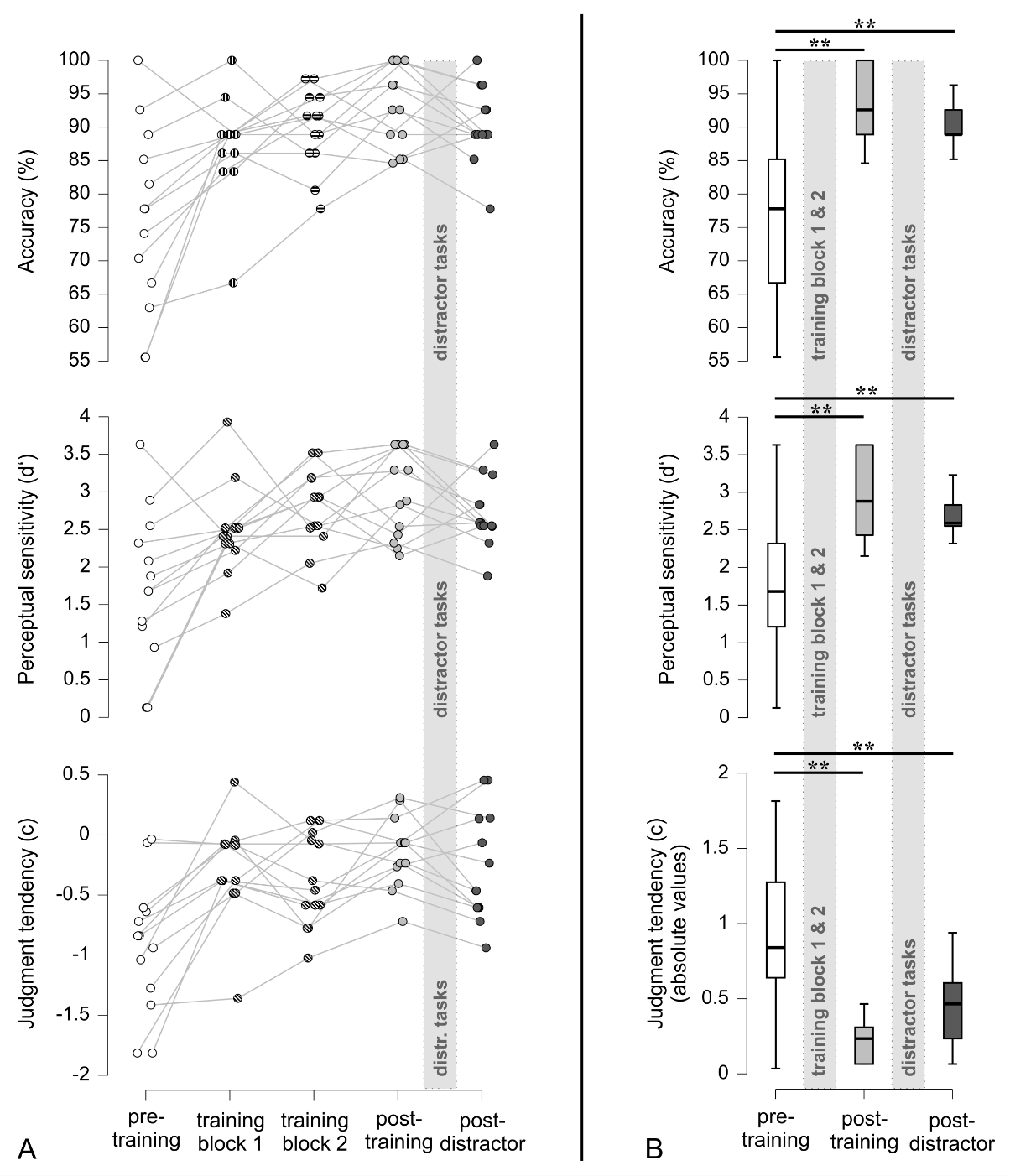


S12 Figure. Distribution of individual data and boxplots for accuracy [%], perceptual sensitivity (d-prime), and judgment tendency (criterion) performance in the Hurdle Task (N=13).

(A) Distribution of the individual data per person across time points for accuracy, perceptual sensitivity and, judgment tendency. (B) Boxplots representing performance at a certain time point (pre-training, post-training, post-distractor). The significance of time point comparisons is indicated. Please note. For judgment tendency, we used absolute values to analyze the deviation from an ideal criterion (which equals 0): The closer to zero the better the performance. Note. * p < .05, ** p < .01 (after Holm–Bonferroni adjustment).
